# Use of bottle of cow's milk in nursery

**DOI:** 10.1186/1939-4551-8-S1-A283

**Published:** 2015-04-08

**Authors:** Isaac Azevedo Tenorio, Aderbal Sabra, Selma Sabra

**Affiliations:** 1 Unigranrio, Brazil

## Introduction

FoodAllergy (FA) has become a common problem in practice for the gastroenterologist and allergists, by increasing its frequency world wide. One cause of this increasing factor is the use of bottle of cow’s milk prior to breast milk. The aim of this work is to add new data for the inappropriate habit of offering a bottle of cow'smilk in the nursery before the breast Milk.

## Material

130 patients with FA colected among the charts of the Brasilian Society of Food Allergy were studied regards the use of bottle fed prior to breast at the nursery station.

Patients were classified according to their clinical and their laboratory tests in patients with mediated IgE FA, non-IgE mediated FA and mixed IgE and non-IgE FA.

## Results

Of the 70 patients with IgE-mediated AA, 44 took the bottle in the nursery before human milk (HM) (62, 85%); of 42 patients with non-IgE, 19 took the bottle in the nursery before the HM (45.23%), and 18 patientswith mixed FA, 8 took the cow's Milk before the HM (44.44%)

## Discussion

The results show that the use in the nursery of the bottle of cow’s milk correlated with the cases of IgE-mediated FA (62.85% and 44.44% versus 45.23%) than the other types of allergies. This finding concurs with the literature data, with respect to that allergen stimuli in the first days of life, induce newborn to produce IgE Food Allergy.

## Conclusion

Early introduction of any other protein in the diet of the newborn before breast milk is associated, at high rates, to IgE FA.

